# Analyzing big datasets of genomic sequences: fast and scalable collection of *k*-mer statistics

**DOI:** 10.1186/s12859-019-2694-8

**Published:** 2019-04-18

**Authors:** Umberto Ferraro Petrillo, Mara Sorella, Giuseppe Cattaneo, Raffaele Giancarlo, Simona E. Rombo

**Affiliations:** 1 Dipartimento di Scienze Statistiche, Università di Roma - La Sapienza, Rome, 00185 Italy; 2 Dipartimento di Ingegneria Informatica, Automatica e Gestionale, Università di Roma - La Sapienza, Rome, 00185 Italy; 3 Dipartimento di Informatica, Università di Salerno, Fisciano (SA), 84084 Italy; 4 Dipartimento di Matematica ed Informatica, Università di Palermo, Palermo, 90133 Italy

**Keywords:** Distributed computing, Apache Spark, k-mer counting, Performance evaluation

## Abstract

**Background:**

Distributed approaches based on the MapReduce programming paradigm have started to be proposed in the Bioinformatics domain, due to the large amount of data produced by the next-generation sequencing techniques. However, the use of MapReduce and related Big Data technologies and frameworks (e.g., Apache Hadoop and Spark) does not necessarily produce satisfactory results, in terms of both efficiency and effectiveness. We discuss how the development of distributed and Big Data management technologies has affected the analysis of large datasets of biological sequences. Moreover, we show how the choice of different parameter configurations and the careful engineering of the software with respect to the specific framework under consideration may be crucial in order to achieve good performance, especially on very large amounts of data. We choose *k*-mers counting as a case study for our analysis, and Spark as the framework to implement FastKmer, a novel approach for the extraction of *k*-mer statistics from large collection of biological sequences, with arbitrary values of *k*.

**Results:**

One of the most relevant contributions of FastKmer is the introduction of a module for balancing the statistics aggregation workload over the nodes of a computing cluster, in order to overcome data skew while allowing for a full exploitation of the underlying distributed architecture. We also present the results of a comparative experimental analysis showing that our approach is currently the fastest among the ones based on Big Data technologies, while exhibiting a very good scalability.

**Conclusions:**

We provide evidence that the usage of technologies such as Hadoop or Spark for the analysis of big datasets of biological sequences is productive only if the architectural details and the peculiar aspects of the considered framework are carefully taken into account for the algorithm design and implementation.

## Background

With the rapid growth of biological sequence datasets and the evolution of the sequencing technologies, many algorithms and software systems commonly used for the analysis of biological sequences are becoming obsolete. For this reason, computational approaches based on frameworks for big data processing started to be proposed in order to deal with problems involving large amounts of biological data [1–5]. Unfortunately, the fundamental domain of alignment-free linguistic and informational analysis of genomic and proteomic sequences, e.g., [6–14], has received yet little attention in this context. In this respect, an important task that is at the hearth of this domain is the collection of *k*-mer statistics, i.e., how many times each sequence of length *k* over a finite alphabet appears in a set of biological sequences, at a genomic scale. Once that such information is available, one can use it to compute many informational and linguistic indices [7, 15], as well as de Bruijn graph assemblers, and error/repeat detection systems.

Recently, the software tool KCH [16] has been proposed for the linguistic and informational analysis of biological sequences based on Hadoop [17] and MapReduce [18]. It allows for an efficient collection and analysis of *k*-mers from a collection of genomic sequences. KCH has been the first tool showing that big data technologies can be superior to highly-optimized shared memory multi-processor approaches, even when considering mid-size problem instances. This latter methodological contribution, combined with results in [19], gives experimental evidence that big data technologies can be extremely pervasive for an effective solution of a broad spectrum of computational problems in the Life Sciences, going from basic primitives to full-fledged analysis and storage pipelines. However, in quantitative terms, that is only a first step towards the acquisition of full knowledge of how big data technologies can affect Computational Biology and Bioinformatics.

In this manuscript, we consider the problem of *k*-mer counting as a case study, and in particular we present a distributed approach for *k*-mer counting that extends the capabilities of KCH and shows better performances. The system is called FastKmer, and has been carefully engineered in order to maximize the potential of Apache Spark [20], the big data framework on which it is based. The result is that, to the best of our knowledge, FastKmer is the fastest distributed system available so far for extracting *k*-mer statistics from large genomic and meta-genomic sequences using arbitrary values of *k*. The approach, devotes particular attention to enforcing a balanced distribution of the workload of *k*-mer statistics aggregation tasks over the nodes of a computing cluster. This same approach may be useful in other scenarios involving notions that are more general than *k*-mers, like *spaced words* and *seeds* (see [21, 22] and references therein). The performance of FastKmer has been evaluated by conducting a comparative experimental analysis with other *k*-mer statistics systems over real-world datasets.

The rest of the manuscript is organized as follows. In the following part of this section, some background for our work is provided. In the Methods section, the algorithm FastKmer is described along with some implementation details. The results of an experimental evaluation of FastKmer, and an improved version of it, are presented in the Results and Discussion section, as well as an experimental comparison with other systems for the collection of *k*-mer statistics. Finally, some conclusions and future directions for our work are outlined in the Conclusions section.

### MapReduce

MapReduce [23] is a paradigm for the processing of large amounts of data on a distributed computing infrastructure. Assuming that the input data is organized as a set of 〈*key*,*value*〉 pairs, the paradigm is based on the definition of two functions, *map* and *reduce*, respectively. Map processes an input 〈*key*,*value*〉 pair and returns a (possibly empty) intermediate set of 〈*key*,*value*〉 pairs. Reduce merges all the intermediate values sharing the same key, in order to form a (possibly smaller) set of values. These functions are run, as tasks, on the nodes of a distributed computing cluster. All the activities related to the management of the lifecycle of these tasks, as well as the collection of the map results and their transmission to the reduce functions, are transparently handled by the underlying framework (*implicit parallelism*), with no burden on the programmer side.

Apache Hadoop is the most popular framework supporting the MapReduce paradigm. It allows for the execution of distributed computations, based on the interplay of two architectural components: YARN (*Yet Another Resource Negotiator*) [24] and HDFS (*Hadoop Distributed File System*) [25]. YARN manages the lifecycle of a distributed application by keeping track of the resources available on a computing cluster, and allocating them for the execution of application tasks modeled after one of the supported computing paradigms. HDFS is a distributed and block-structured file system designed to run on commodity hardware and able to provide fault tolerance through data replication.

Since their introduction, both MapReduce and Hadoop have become a cornerstone of big data processing. The key for their success is that the MapReduce-based programming interface supported by Hadoop provides developers with a quite convenient environment to code effective applications, allowing them to focus more on the specific task at hand, rather than on other issues such as synchronization and process-to-process communication, as opposed to what traditional, low-level primitives such that provided by MPI Standard (Message Passing Interface [26]) or its ancestor, PVM (Parallel Virtual Machine [27]), allowed for. Indeed, within such programming environments, concurrency must be explicitly handled by the programmer and the running program strongly depends on the physical network topology. In the realm of Bioinformatics, this point is well illustrated in the Magellan Final Report [28] regarding the collection of *k*-mer statistics in large meta-genomic datasets: MPI solutions would work, but it is much more convenient to use Hadoop and MapReduce, also considering the availability of higher level tools like Pig [29, 30].

In the remaining part of this section, first we recall some basic notions on Apache Spark, which is central for the approach presented here. Then, we provide a summary of the main approaches for *k*-mers counting based on big data technologies.

### Apache Spark

Spark is a fast and general distributed system for cluster computing on big data. It consists of two main blocks: a programming model that creates a dependency graph, and an optimized runtime system which uses this graph to schedule work units on a cluster, and also transports code and data to the *worker* nodes of this cluster where they will be processed by *executor* processes.

**Resilient Distributed Datasets** At the core of the Spark programming model is the *Resilient Distributed Dataset* (RDD) abstraction, a fault-tolerant, distributed data structure that can be created and manipulated using a rich set of operators. Programmers start by defining one or more RDDs through *transformations* of data that originally resides on stable storage or other RDDs (e.g., map, filter or reduce).

Apart from the internal cluster manager, Spark applications can be run also on external cluster managers like Hadoop YARN [24] or Mesos [31]. Moreover, a Spark application can be run over a distributed file system, e.g., HDFS [25]. This allows each worker node of a cluster to read input data and to write output data using a local disk rather than a remote file server.

**Partitions, parallelism, and shuffling**

By default, Spark tries to read data into an RDD from the nodes that are close to it. Since Spark typically accesses distributed data, to optimize transformation operations, it creates partitions to hold the data chunks. The number of partitions of an RDD reflects the degree of *parallelism* (number of tasks) employed by Spark while processing it. When an RDD is created by loading files from HDFS, its number of partitions is equal to the number of input splits of the original file on HDFS. The size of an input split depends on the *block size*, a configurable parameter of the MapReduce ecosystem.

The Spark mechanism for redistributing data across partitions is called *shuffling*. It occurs when certain transformations, such as groupByKey or reduceByKey, are issued on an RDD and cause moving data across different processes or over the wire (between executors on separate nodes). An RDD that is obtained via a shuffle transformation of another RDD will inherit its number of partitions. However, as far as choosing a “good” number of partitions is of concern, what is typically desired is to have at least as many partitions as the number of cores. A way to influence this choice is by specifying a custom value for the spark.default.parallelism property. Another option, consists of introducing a custom *partitioner*. Partitioners are objects that define how elements of a key-value RDD are partitioned by key. The Spark default partitioner (i.e., HashPartitioner) chooses the partition where to map an element as the Java’s Object.hashCode value of its key (modulo number of partitions), or 0 for negative hashes. It is also possible to implement a custom partitioner class defining a custom method for assigning keys to partitions. This feature is useful when, for some reason, the default partitioner causes RDD data to be unevenly distributed across partitions.

### Big Data based approaches for the analysis of biological sequence datasets: the special case of *k*-mers counting

The modern high-throughput technologies produce large amounts of sequence collections of data, and several methodologies have been proposed for their efficient storage and analysis [15, 32]. Recently, approaches based on MapReduce and big data technologies have been proposed (see, e.g., [2], and [3] for a complete review on this topic). An important issue in this context is the computation of *k*-mer statistics, that becomes challenging when sets of sequences at a genomic scale are involved. Due to the importance of this task in several applications (e.g., genome assembly [33] and alignment-free sequence analysis [15, 32]) many methods that use shared-memory multi processor architectures or distributed computing have been proposed.

The basic pattern followed by most of these methods is to maintain a shared data structure (typically, a hash table) to be updated according to the *k*-mers extracted from a collection of input files by one or more concurrent tasks. When memory is not enough to maintain all the extracted *k*-mers, these can be organized in disjoint partitions and temporarily saved on file without aggregation. Then, they will be loaded in memory one partition at time and summed to return the definitive *k*-mer statistics.

Here, we provide a summary of the main techniques proposed for *k*-mers counting in the Bioinformatics scenario, organized in two main categories: those designed to work on shared memory and/or multi-processor systems, and those implemented for distributed systems (the interested reader can refer to an extensive survey in [16]).

**Shared memory, multi-processor systems.** MSPKmerCounter [34] introduces a disk-based approach where consecutive *k*-mers are not saved individually but first compressed to a single *superkmer*. This solution leads to a significant reduction in the amount of data to be temporarily saved on disk and, then, recovered to memory, thus allowing for a significant performance boost with respect to other algorithms. The notion of minimizer has been refined in KMC2 [35] and, later, in KMC3 [36] with the introduction of *k*-mer *signatures*. These are a specialization of minimizers and are built with the idea of discouraging an extremely imbalanced partitioning of superkmers among the different bins while keeping the overall bins size as small as possible. An additional contribution provided by these systems is in the counting phase. Input superkmers are broken into (*k, x*)-mers, a compact representation of a sequence of *k*-mers whose length is ≤(*k*+*x*), and sorted efficiently using a parallel version of radix sort [37].

**Distributed systems.** The applicability and scalability of multi-processor shared-memory architectures is inherently constrained by architectural factors, such as the maximum number of processing cores on a processor, and the maximum amount of memory on a single hosting machine. Distributed systems allow to overcome these limitations. Indeed, the availability of an arbitrary number of independent computation nodes makes it possible to virtually extend to any size the data structure used to keep the *k*-mer statistics in memory, while using the network as a temporary buffer between the extraction phase and the aggregation phase.

This is the approach followed by Kmernator [38] and Kmerind [39]. Both these tools are developed as MPI-based parallel applications and are able to handle data sets whose size is proportional to the overall memory of the MPI-based system where they are run. However, the development and management of an in-house MPI-based supercomputing facility is usually very complex and expensive.

BioPig [40] is an Hadoop-based analytic toolkit for the processing of genomic data. It has been developed as an extension of the Pig language that, in turn, offers a set of data querying and transformation primitives that are translated into MapReduce jobs. BioPig includes a module, called pigKmer, that allows to extract and count the *k*-mers existing in a set of sequences. Each sequence is split into several blocks saved on the different nodes of a distributed system, with each block being processed by a distinct task. The *k*-mers extracted in this way are then aggregated, using a reduce operation, and finally counted. An alternative distributed *k*-mers counter is the one provided by ADAM [41], a Spark-based toolkit for exploring genomic data, which follows the same application pattern of BioPig. The algorithmic approach of these two systems is somewhat simplistic, so they are able to process very large genomic sequences but at the expense of very poor resource utilization.

The first and, to date, the only distributed system able to extract efficiently *k*-mer statistics from large collections of genomic sequences, with *k*≤31, is KCH [16]. It is a distributed system, based on MapReduce and Hadoop, which follows a two-level aggregation strategy. In particular, it first partitions the universe of possible *k*-mers into a fixed number of bins (291, by default) and, then, it extracts the *k*-mers counts from a collection of input sequences in two stages of alternate map and reduce tasks. In the first stage, each map task creates a distinct hash table for each bin and updates them with the statistics of the *k*-mers extracted from a chunk of the input sequences. At the end of this stage, each map task returns its collection of hash tables holding the partial *k*-mer counts. During the second stage, all the hash tables corresponding to the same bin are aggregated by a reduce task and the result is saved on file. This strategy is able to significantly reduce the communication overhead between the different nodes of the system, thus allowing for execution times that are up to 100× faster than those of BioPig, when run on fairly large sequences.

## Methods

In this section we describe the core concepts and the main design aspects behind our algorithm, FastKmer.

### Basics

Let *Σ* be an alphabet and *S* be a finite set of collections of sequences over *Σ*. A *cumulative statistics* collects how many times each of the *k*-mers in *Σ*^*k*^ appears in the collections of sequences in *S*. Here *Σ*={*A, C, G, T*} and *S* is a collection of genomes or meta-genomes.

Algorithms that compute *k*-mer statistics usually have a common structure: they first process the sequences in *S* from left to right in order to extract all *k*-mers and, then, they perform aggregation and evaluation. A naive implementation, such that all single *k*-mers are extracted in a sliding window fashion, is highly redundant in space. Indeed, for an input length of *n* characters, generating all *k*-mers determines an unfolded sequence of (*n*−*k*+1)·*k* symbols. Since, by definition, consecutive *k*-mers along a sequence share *k*−1 symbols, it would be beneficial to have a compressed representation of them, where all contiguous *k*-mers are stored in a compact sequence. Yet unfortunately, to be able to collect the statistics, especially in a distributed setting where different portions of the input data are processed by physically separated machines, we need a way to keep together all instances of each unique *k*-mer for the evaluation phase. A clever solution to this problem is based on the notion of minimizers [34, 42, 43].

**Minimizers** Given a *k*-mer *s*, a *minimizer* of *s* is a word of length *m* (with *m* fixed a priori) occurring in *s*. Usually many consecutive *k*-mers have the same minimizer and, therefore, can be compressed into a sequence of more than *k* symbols, a *superkmer*, significantly reducing the redundancy.

Minimizers may be used to partition *k*-mers into multiple disjoint sets, as well as retaining adjacent *k*-mers in the same partition: superkmers can be distributed into different bins according to their related minimizer ensuring that all the corresponding instances of a *k*-mer will appear in the same bin.

### *k*-mer statistics collection on Spark: the FastKmer algorithm

Here FastKmer is described, focusing also on the engineering aspects which make it more efficient with respect to its competitors (e.g., KCH).

### Design overview

**Fig. 1:**
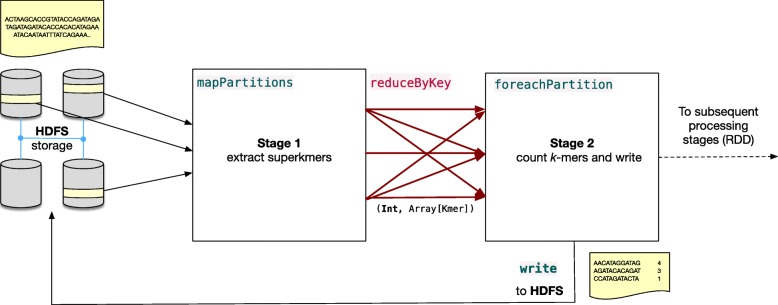
Stages of the pipeline implementing FastKmer

The FastKmer algorithm is implemented on the Spark pipeline described in Fig. 1. The core of the pipeline consists of two main stages, as well as a preliminary stage responsible of fetching the input dataset, that is a FASTA/FASTQ file [44, 45], from HDFS storage, and delivering all of its blocks to the first stage of the pipeline (leftmost portion of Fig. 1). The first stage performs the extraction of superkmers. The second stage computes and collects the *k*-mer statistics. Both stages are described in detail in the following.

#### First stage: extracting superkmers

**Fig. 2:**
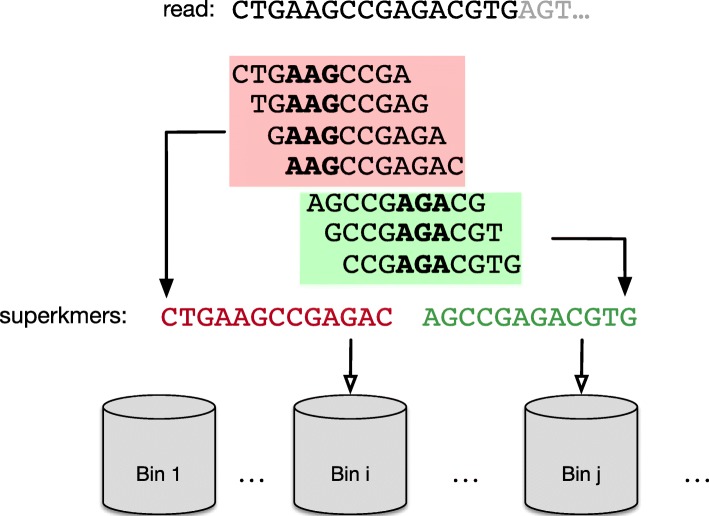
Extraction of superkmers from input sequences, using signatures (*k*=10,*m*=3)

To address the problem of redundancy, the first stage of our approach processes all input sequences in a way to guarantee a degree of compression: sequences are broken into superkmers using their corresponding minimizers which are in turn used to implement a binning mechanism. In particular, FastKmer adopts a slightly different notion that is the one of *signatures* [35], i.e., canonical minimizers of length *m* (a tunable parameter) that do not start with *AAA* nor *ACA*, neither contain *AA* anywhere except at their beginning. A toy example of splitting a sequence into superkmers using signatures is depicted in Fig. 2. This is a variant of the Minimum Substring Partitioning (MSP) technique [46, 47].

##### From signatures to bins

Superkmers having a given signature *s* are then mapped to one of a set of *B* bins (a parameter) using a shift-based integer hash function, thus aiming at a uniform distribution of superkmers in processing units for the subsequent phase.

The output of the first stage is therefore a sequence of bins, where each bin is described by an integer key in the range {1,…,*B*} and holds a sequence of superkmers. Then, bins originating from different distributed workers are automatically aggregated by Spark based on their key in an intermediate phase before the next stage (red shuffling phase in Fig. 1).

#### Second stage: partitioned counting

The second stage is responsible of the counting phase: due to the signature-based binning process, all instances of a given *k*-mer are guaranteed to reside in the same bin. Therefore, each bin is processed independently and all the *k*-mers contained therein are inserted in a hash table, that also maintains their relative counts. After processing each bin, the table is traversed, and counts are saved on HDFS.

#### Implementation details

FastKmer has been implemented as a Spark application using Scala [48]. a programming language that combines the object-oriented paradigm of Java with aspects from functional programming such as local type inference, functional combinators and immutable data structures. This choice allows for a concise and efficient implementation of the FastKmer algorithm while ensuring the best interoperation possible with Spark (which is natively implemented in Scala as well).

Input sequences are read by FastKmer using FASTdoop [49].

##### Compressed *k*-mer encoding

FastKmer represents *k*-mers (and likewise, superkmers) of arbitrary length using an Array[Long], where information is encoded by a binary representation. Therefore, since the alphabet of valid nucleotides consists of four items, as *Σ*={*A, C, G, T*}, a string over *Σ*^*k*^ can be encoded using only two bits per symbol. Representing the original sequence using a concatenation of 2-bit numbers, by means of binary operations, allows for a space compression of about 75%, since each character of a *k*-mer needs two bits rather than eight (corresponding to the Java String implementation, and ignoring its additional overhead). The impact of this choice is is particularly relevant when considering the network traffic involved in the shuffling stage of Fig. 1. Since the Long type uses 64 bits, each item in the array can store up to 31 symbols (as opposed to 32, since the most significant bit is reserved for sign); the last item of the array is padded with leading zeros (and this information is kept together with the encoded *k*-mer).

## Results and discussion

Here we describe the results of an experimental analysis that shows how different choices of the parameters, and Spark-related configurations may result in different performances of FastKmer.

### Setup

**Testing platform** The experiments have been performed on the Microsoft Azure Cloud infrastructure. In particular, a 8-node Spark 2.1.0 cluster has been deployed into HDInsight, Microsoft’s cloud distribution of the Hadoop ecosystem (Hadoop 2.7.3), based on the Hortonworks Data Platform. Two cluster nodes act as head nodes, and are equipped with an 8-core 2.4 GHz Intel Xeon E5-2673 v3 processor and 28GB of RAM each. Furthermore, the cluster has other six worker nodes, each with two 8-core 4.78 GHz Intel Xeon E5-2673 v3 processors for a total of 16 cores, 112GB of RAM and a 800GB local SSD disk, and an overall disk capacity of 4.8TB.

All Spark jobs have been configured to use 2 cores per executor, the number of executors, instead, varies according to the specific experiment performed.

**Datasets** For the experimental validation, we have used the metagenomic dataset described in [50]. In particular, the *SRR*094926 run of the *SRP*004875 SRA study (available on the NCBI short read archive) has been considered, for a total space occupation of about 125GB (FASTA format). Furthermore, another dataset containing only the first 32GB of this run has also been considered. See Table 1 for summary information.

**Table 1:** Number of distinct and total *k*-mers for our datasets

	*k*=28		*k*=55	
kmers	32GB	125GB	32GB	125GB
Distinct	12,551,234 K	37,337,258 K	14,203,028 K	47,830,662 K
Total	22,173,612 K	86,674,803 K	18,722,642 K	73,209,044 K

### Experimental evaluation

This section presents an experimental study of different configurations and parameters, as well as of their main implications on the performances of FastKmer.

**Values of*k*.** For all experiments, we examine the running time performance of the *k*-mer statistics collection task on our datasets for two different reference values of *k*: 28 and 55. Following the choice of [35], these two values have been chosen as examples that reflect both the case where each *k*-mer can be stored using a single Long variable, as well as the case where it requires more variables.

**Signature length.** Preliminary experiments have been performed in order to tune the signature length parameter *m* (data not shown but available upon request). The result is that small values of *m* increase the probability that consecutive *k*-mers share the same minimizer and thus reduces the I/O cost at the end of the first stage. However, if too small, it might render the distribution of partition sizes skewed and the largest partition might not fit in memory. On the other hand, a large value of *m* will make the distribution of partition sizes more balanced at the cost of a higher redundancy (with no compression for *m*→*k*).

The assignment which empirically yields, on average, the best performance on the considered datasets is *m*=10. This is in line with the results in [35, 46] on datasets of comparable characteristics.

**Number of bins.** The number of bins *B* used for the signatures binning scheme is considered. At the starting of the second stage, each partition contains a number of bins to be processed. Having few bins decreases the overall memory management overhead to be paid at the beginning and at the end of the processing of each bin. As a downside, having few, very large bins might require an amount of memory exceeding the one available to a worker process. On the other hand, a larger number of bins allows for a better granularity of the distributed execution and reduce memory requirements for each worker process, as each bin can be processed independently. However, in such a case, there is an increased memory management overhead for each worker process.

**Spark parallelism.** A Spark-specific parameter which may have an impact on the running time and cluster usage, is the Spark *parallelism level* (*p*). This parameter corresponds to the number of tasks that are spawned by Spark (as well as the number of partitions). Its choice has a side effect on the number of bins mapped to partitions: if bin numbers are uniformly spread, each task will receive a number of bins that tends to *B*/*p*.

**On large bins.** As previously stated, when using the minimizer-based approach, the distribution of superkmers associated to signatures can be very uneven, with particularly frequent signatures tending to have a very large fraction of superkmers. This is mainly driven by the minimizer-based scheme: the distribution of superkmers associated to signatures can be very uneven, with low lexicographic signatures, tending to have a very large fraction of superkmers, compared to the rest. This is partly mitigated by the choice of signatures within a suitably filtered sets of canonical minimizers that do not start with common prefixes, and by the hash-based mapping of signatures to bins. Nevertheless, since the scheme is data-oblivious, it might still produce large bins. In our distributed setting, this is particularly relevant because they can introduce bottlenecks where the running time is impacted by a few number of workers that take more time than the others, thus leading to a non-optimal utilization of the cluster.

**Fig. 3:**
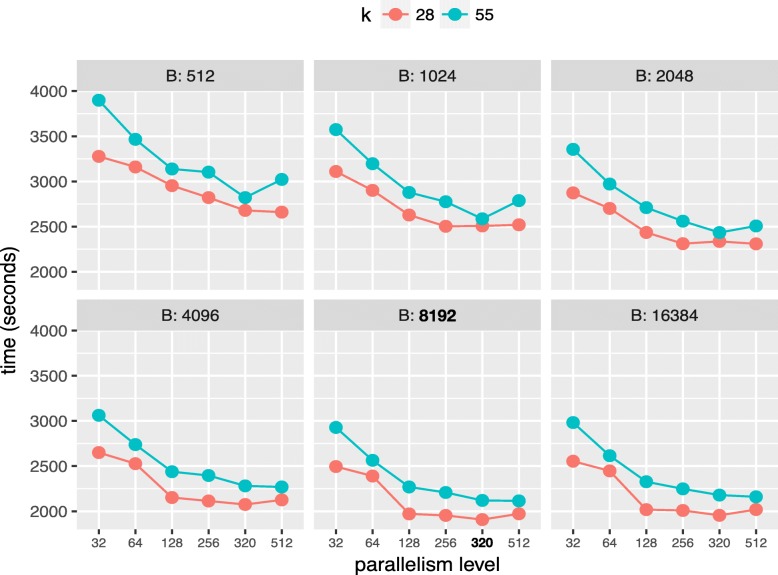
Algorithm execution times on the 32GB dataset with *k*=28,55 and an increasing number of bins (B) and parallelism level. Best combination shown in bold

**Experimental results.** Figure 3 shows the running time of FastKmer when run with 16 executors, (for a total of 32 workers), using various values of *B* corresponding to powers of two between 512 and 16.384, and a parallelism levels ranging from 32 to 512 (corresponding, respectively, to 1 and 16 average tasks per core). As per the number of bins, performances improve consistently for values of *B* up to 8192, after which we have no improvement (also for higher values of *B*, not plotted for legibility). The x-axis shows the parallelism *p*: again, for both values of *k*, we see a performance increase when we raise *p* up to 320 tasks. No improvement, if not a slight deterioration, is noticeable for higher values of *p*. This is expected: while higher parallelism tends to better spread large bins across many partitions, conversely, more tasks determine more scheduling overhead for their start and finalization. Spark tuning guidelines, [51], suggest setting a parallelism level that is in the range of 2−3 tasks per core: this choice of *p* leads to 10 tasks per core in our test instance (32 workers), suggesting a possible heavy scheduling-related overhead.

Intuitively, increasing both values of *B* and *p* mitigates the big bins problem, since: *(i)* mapping signatures to more bins means potentially big signatures to be spread over a larger number of bins, *(ii)* increasing parallelism allows to split big bins as they are more granular and distributed across more tasks. This does not fully remedy the fact that bins can have very different sizes.

A further inspection of the distribution of the single task running times for low values of *B* and *p* shows that some tasks take much longer than others (peaking at as much as 50% of cluster underutilization, for some configurations). With larger values of *B* and *p*, and in particular for the best configuration *B*=8192 and *p*=320, the problem is indeed mitigated, yet still we have a single task running at the end of the job for about 5% of the running time.

Another problem is related to the fact that the optimal values of *B* and *p* ultimately depend on the dataset. We wish to have a solution that allows for a degree of adaptability of the algorithm to dataset variability, and, more generally, exhibiting better load balancing guarantees. In the next section, we explore a promising direction of improvement to address these issues.

#### Coping with data skew: a multiprocessor scheduling inspired partitioner

As previously stated, the unbalanced partitioning of bins resulting from our experiments is mainly driven by the minimizer-based scheme. Bins exacerbate this fact even more, as they contain multiple signatures, (and possibly many of such “big” ones), with the consequence that few larger bins lead some workers to have a much longer running time than the others. The standard partitioner of Spark does not come into rescue, as it maps bin ids to partitions following their hashCode, and therefore cannot take into consideration their size.

The necessity of achieving a balanced distribution of the workload induced by bins while taking into account the actual number of available processing units can be framed as an instance of the more general *Multi-Processor Scheduling (MPS)* problem [52]. In this problem, the input consists of *t* identical machines and *n* jobs, where each job has a processing time greater than zero. The goal is to assign jobs to machines so as to minimize the maximum load of a machine (i.e., the sum of the processing time of its jobs) which, as all the machines operate in parallel, can be seen as the actual schedule time. Computationally, MPS is NP-Hard, therefore FastKmer resorts to a simple heuristic: the *Longest Processing Time* (LPT) algorithm. LPT proceeds by first sorting jobs by processing time and then by assigning each of them to the machine with the earliest end time (i.e., lowest load) so far. This algorithm achieves an upper bound of $\left (\frac {4}{3} - \frac {1}{3t} \right) OPT$ [53].

In this setting, jobs correspond to bins, and their processing time is estimated as the number of *k*-mers contained in the corresponding bin (later, referred to as *bin size*), and the number of machines is assumed to be the number of partitions. From the FastKmer viewpoint, the integration of this scheduling algorithm requires two main modifications to the original pipeline. At the beginning of the computation, a new preliminary stage is run to derive an estimation of bin sizes by examining a sample of the input data (whose size can be specified, defaulting to 1%). This estimation is then used to compute a schedule using LPT. In turn, the resulting schedule is used by a custom partitioner replacing the original one available with Spark for mapping bins to partitions at the end of the first stage.

##### Granularity of working units.

In order to further mitigate the “big bins problem”, we also take into consideration a variant of the partitioning scheme, that instead of bins, uses the signature value itself to implement the binning process. In our multiprocessor scheduling analogy, the set of superkmers belonging to a signature represents a job. This choice achieves two major benefits: *(i)* it allows the removal of a parameter (*B*) which should be, otherwise, optimized, *(ii)* it allows for the finest granularity of work units for the second phase of the task, that will prove to be particularly convenient for our custom-partitioner based implementation, as shown in the following experimental section.

##### Custom partitioning results.

**Fig. 4:**
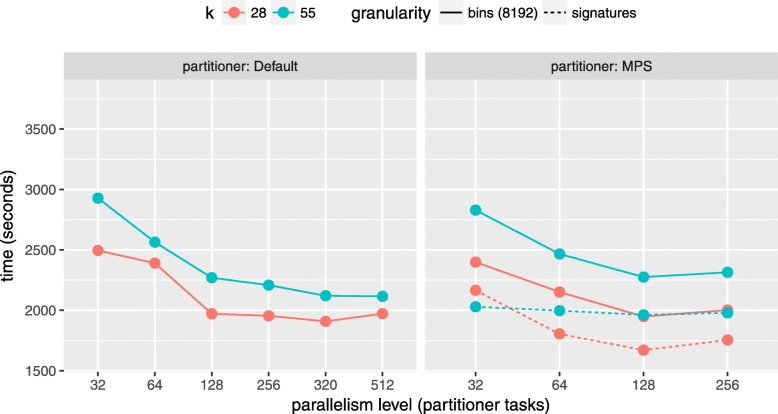
Comparison of execution times of FastKmer on the *32GB* for *k*∈{28,55}, using default (left) or custom (right) MPS-based partitioning method, for an increasing parallelism level. The number of bins is set to 8192. For the custom partitioning scheme the performance of the signature granularity is also shown, marked with a dashed line

Figure 4 reports a running time comparison between an implementation of FastKmer using the default Spark partitioner (left), and another one implementing our custom multiprocessor scheduling-based partitioning scheme (right) run on our 32*GB* dataset. As for our custom partitioner, it further compares two different granularities for the work units: *bins* and *signatures* (solid vs dashed lines). For the bins granularity (*B*=8192, resulting from the previous analysis), the impact of the custom partitioner is moderate. For the signatures choice, instead, the improvement of the custom partitioner has a consistently higher margin, suggesting an important impact of the imbalanced signatures distribution. On a related note, it can be noticed that the improvement is starting at the lowest level of parallelism (1 task per CPU core), and increases up to 128 total tasks (4 tasks per CPU core). After 128 tasks we see no improvement: this is also expected as the goodness of a LPT schedule decreases with higher number of machines with respect to the optimal solution (in accordance to LPT bounds with respect to the optimum solution). Based on these results, our default implementation makes use of our custom partitioning scheme, with signature-based binning.

### Comparative experimental analysis

Here an experimental comparison of FastKmer against other big data systems for the extraction of *k*-mer statistics is presented. For each system described in the Background Section, the runtime configuration suggested by their respective authors has been adapted to the considered testing platforms.

First, we have performed a comparison between our system and other distributed k-mer counting processing frameworks reported in the Background Section, on our experimental platform, considering both the 125GB and 32GB datasets while using *k*=28 and *k*=55 and 32 total workers. The corresponding results are shown in Table 2. We have not been able to run ADAM on our testing platform because of memory issues. A further investigation revealed that this system extracts *k*-mers from an input FASTA file by first converting it into another format through an operation that requires the whole file to be loaded in the main memory of the driver process. This approach, apart from being extremely inefficient, prevents the system to work when the driver process has not enough memory to fulfill this operation (like in our case).

**Table 2:** Running time (minutes) for various distributed *k*-mer counting algorithms, with a time limit of 10 hours

	*k*=28		*k*=55	
Algorithm	32GB	125GB	32GB	125GB
FK	23	82	38	119
KCH	28	196	**–**	**–**
BioPig	122	Out of time	450	Out of time
ADAM	Out of mem	Out of mem	Out of mem	Out of mem

Dash symbols represent combinations where the value of *k* is not supported by the algorithm

As expected, the performances of BioPig are considerably lower than those of FastKmer and KCH, taking more than 10 hours to complete, on the 125GB dataset. Indeed, the lack of any aggregation strategy during the *k*-mers extraction phase and the choice of a standard character-based encoding for the extracted *k*-mers increases significantly the amount of data to be moved from the extraction phase to the evaluation phase, thus putting a heavy burden on the overall execution time of this system.

We now turn to the case of KCH. We recall that this system has been developed to only support values of *k* smaller than 32. As for the case of *k*=28, we notice that FastKmer is about 20% faster than KCH when processing the 32GB dataset. This difference becomes even more significant when considering the 125GB dataset. Here, FastKmer is about two times faster than KCH. To explain this, consider that KCH aggregates *k*-mers in bins at a much coarser level and that it lacks a scheduling strategy able to ensure an even distribution of the workload among the nodes of the underlying distributed system.

#### Scalability analysis

In this section, we present a scalability analysis, with respect to cluster scale and dataset sizes. After the preliminary analysis among the distributed framework, performed in the previous sections, we selected the best performing ones: FastKmer and KCH.

**Fig. 5:**
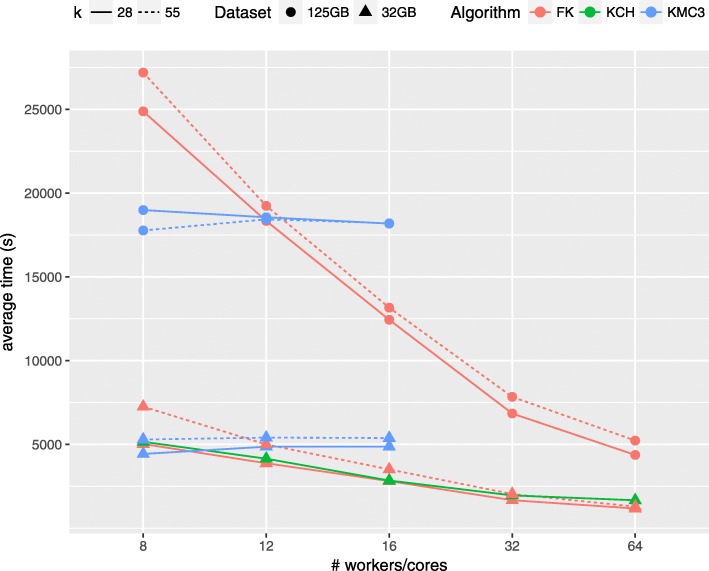
Running time comparison of FK and other *k*-mer counting algorithms for various values of *k* and an increasing number of workers

Figure 5 shows the running time comparison of the two systems for various values of *k* (except for KCH, that supports only values up to *k*=31). From Fig. 5 we see that FastKmer outperforms KCH in terms of running time, for all number of workers.

For completeness, we also compared the performance of FastKmer and KCH to those of the fastest multi-threaded *k*-mers counting system, KMC3 [54], by running the latter on a single node using an increasing number of processing cores (up to 16 cores, the maximum available on a single nodes). From the results depicted in Fig. 5, KMC3 is the fastest system when using a small number of cores, whereas its execution time remains approximately the same for increasing number of workers. This is in line with the literature, as it has already been observed [16] that the performance of this system does not improve when considering a large number of cores.

Overall, FastKmer outperforms previous approaches in terms of running time, showing to scale better for an increasing number of workers on both datasets and values of *k*.

#### Profiling Spark

To conclude, we deepen the analysis on Spark performance overheads, reporting a breakdown of the execution times of FastKmer when run on the *32GB* dataset using an increasing number of workers. The execution time of a Spark task can be broken down into *Scheduler Delay, Deserialization Time, Shuffle Read Time (optional), Executor Runtime, Shuffle Write Time (optional), Result Serialization Time and Getting Result Time* [55]. This information has been collected out of FastKmer runs by collecting the performance metrics readable from Spark Event Logs (except for the Scheduler Delay, which has been calculated from other available metrics, following Spark UI code)

Tables 3 and 4 contain task performance metrics for the two main stages of FastKmer (Fig. 1): the *k*-mers extraction phase (Stage 1) and the *k*-mer counting phase (Stage 2). The preliminary stages that implement the multiprocessor scheduling partitioning schemes have been omitted, as their compute time is negligible with respect to the overall processing, accounting only for a few seconds.

**Table 3:** Running time breakdown, in seconds, of the two FastKmer stages on the *32GB* dataset with *k* fixed to 28 and a decreasing number of executors

	64 Workers	32 Workers	16 Workers	8 Workers
	Stage 1			
Scheduler Delay time	0.07	0.08	0.1	0.26
Executor Deserialization time	0.93	1.01	2.15	3.98
Executor Compute time	351.4	580.9	1112.51	2655.48
Shuffle Read time	0	0	0	0
Shuffle Write time	1.22	2.33	4.59	10.42
Shuffle Read local (MB)	0	0	0	0
Shuffle Read remote (MB)	0	0	0	0
Shuffle Write (MB)	504.7	1009.5	2018.7	4542
	Stage 2			
Scheduler Delay time	0.08	0.14	0.07	0.11
Executor Deserialization time	0.19	0.44	1.05	1.82
Executor Compute time	773.52	868.59	1648.76	3859.24
Shuffle Read time	0.06	0	0	0.01
Shuffle Write time	0	0	0	0
Shuffle Read local (MB)	15.6	62.5	250.6	1125.9
Shuffle Read remote (MB)	484.4	937.9	1749.9	3375.3
Shuffle Write (MB)	0	0	0	0

The table reports also the size, in megabytes, of the corresponding read and write shuffles

**Table 4:** Running time breakdown, in seconds, of the two FastKmer stages on the *32GB* dataset with *k* fixed to 55 and a decreasing number of executors

	64 Workers	32 Workers	16 Workers	8 Workers
	Stage 1			
Scheduler Delay time	0	0.1	0.1	0.2
Executor Deserialization time	0	0.4	0.8	2.7
Executor Compute time	293.4	569.7	1152.8	2575.2
Shuffle Read time	0	0	0	0
Shuffle Write time	0.8	1.7	3.3	7.4
Shuffle Read local (MB)	0	0	0	0
Shuffle Read remote (MB)	0	0	0	0
Shuffle Write (MB)	504.7	1009.5	2018.7	4542
	Stage 2			
Scheduler Delay time	0	0	0.1	0.1
Executor Deserialization time	0.2	0.44	0.4	1.4
Executor Compute time	1083	1171.2	2060.2	4556.4
Shuffle Read time	0	2.3	0	0
Shuffle Write time	0	0	0	0
Shuffle Read local (MB)	15.6	62.5	250.6	1125.9
Shuffle Read remote (MB)	484.4	937.9	1749.9	3375.3
Shuffle Write (MB)	0	0	0	0

The table reports also the size, in megabytes, of the corresponding read and write shuffles

The metrics report, for each stage, the sum of average values for all tasks in groups of (#executors × #executor cores), over each round, up to the parallelism level.

Unfortunately, the *Shuffle Read Time* obtainable by the performance metrics is actually only the *blocking time* during the shuffle reads (also called *Fetch Wait Time*), i.e., the time a thread has to wait for another read to finish to acquire the lock on a shuffled block. The time to actually fetch the block from the shuffle source executor is included in the Executor Compute Time (which may be from disk, if the shuffle block cache spilled). No metric exists for the read time of a remote block during a shuffle read. For this reason, to give a sense of the Shuffle Read overhead, we have also reported the local and remote Shuffle Read size (in MB). For completeness, we have included the Shuffle Write size as well. As can be clearly seen by the two tables, the performance metrics scale linearly with the number of workers.

## Conclusions

It is worth to remark that the advantages of technologies like Hadoop or Spark for the analysis of big data come at a cost. A naive usage of these technologies may bring to solutions that, although being able to run on big data, are inefficient.

In this paper, we have presented FastKmer, an efficient system for the extraction of *k*-mer statistics from large collection of genomic and meta-genomic sequences using arbitrary values of *k*. FastKmer succeeds in being, to the best of our knowledge, the fastest *k*-mer statistic distributed system to date, not only because it implements a clever algorithm for the extraction and the aggregation of *k*-mers, but even because it has been purposely engineered and tuned so to extract the most from the underlying Spark framework. This is especially the case of the different strategies that we developed for the distribution of the *k*-mers aggregation workload over a Spark cluster, and that can be used as well in more general Bioinformatics application scenarios.

As a future direction, we observe that the internal architecture of FastKmer has been conceived so as to make it easy to integrate its workflow in more complex data processing pipelines. For instances, we cite the case of distributed alignment-free algorithms. These could use FastKmer as a sub-pipeline to extract the *k*-mers from each sequence of a collection for later comparison.

## Abbreviations

HDFS: Hadoop distributed file system
LPS: Longest processing time
MPI: Message-passing interface
MPS: Multi-processor scheduling
MSP: Minimum substring partitioning
PVM: Parallel virtual machine
RDD: Resilient distributed dataset
YARN: Yet another resource negotiator
